# Boosting with intranasal dendrimeric Aβ1–15 but not Aβ1–15 peptide leads to an effective immune response following a single injection of Aβ1–40/42 in APP-tg mice

**DOI:** 10.1186/1742-2094-3-14

**Published:** 2006-06-05

**Authors:** Timothy J Seabrook, Liying Jiang, Katelyn Thomas, Cynthia A Lemere

**Affiliations:** 1 Center for Neurologic Diseases, Brigham and Women's Hospital, Harvard Medical School, Boston, MA 02115, USA

## Abstract

**Background:**

Immunotherapy for Alzheimer's disease (AD) is emerging as a potential treatment. However, a clinical trial (AN1792) was halted after adverse effects occurred in a small subset of subjects, which may have been caused by a T cell-mediated immunological response. In general, aging limits the humoral immune response, therefore, immunogens and vaccination regimes are required that induce a strong antibody response with less potential for an adverse immune response.

**Method:**

In the current study, we immunized both wildtype and J20 APP-tg mice with a priming injection of Aβ1–40/42, followed by multiple intranasal boosts with the novel immunogen dAβ1–15 (16 copies of Aβ1–15 on a lysine tree), Aβ1–15 peptide or Aβ1–40/42 full length peptide.

**Results:**

J20 APP-tg mice primed with Aβ1–40/42 subcutaneously and subsequently boosted intranasally with Aβ1–15 peptide did not generate a cellular or humoral immune response. In contrast, J20 APP-tg mice boosted intranasally with dAβ1–15 or full length Aβ1–40/42 produced high levels of anti-Aβ antibodies. Splenocyte proliferation was minimal in mice immunized with dAβ1–15. Wildtype littermates of the J20 APP-tg mice produced higher amounts of anti-Aβ antibodies compared to APP-tg mice but also had low T cell proliferation. The anti-Aβ antibodies were mainly composed of IgG2b and directed to an epitope within the Aβ1–7 region, regardless of the immunogen. Examination of the brain showed a significant reduction in Aβ plaque burden in the J20 APP-tg mice producing antibodies compared to controls. Biochemically, Aβ40 or Aβ42 were also reduced in brain homogenates and elevated in plasma but the changes did not reach significance.

**Conclusion:**

Our results demonstrate that priming with full length Aβ40/42 followed by boosting with dAβ1–15 but not Aβ1–15 peptide led to a robust humoral immune response with a minimal T cell response in J20 APP-tg mice. In addition, Aβ plaque burden was reduced in mice producing anti-Aβ antibodies. Interestingly, wildtype mice produced higher levels of anti-Aβ antibodies, indicating that immune tolerance may be present in J20 APP-tg mice. Together, these data suggest that dAβ1–15 but not Aβ1–15 peptide may be useful as a boosting immunogen in an AD vaccination regime.

## Background

Alzheimer's disease (AD) is a devastating disease characterized by a progressive deterioration in cognitive abilities, eventually leading to severe dementia. Pathologically there is localized deposition of cerebral β-amyloid (Aβ) protein, neuritic plaques, glial activation, neurofibrillary tangle formation, and neuronal loss [1]. The cause of AD is still an area of debate however, there is accumulating epidemiologic, pathologic and genetic evidence that Aβ has a pivotal role in the pathogenesis of AD, suggesting that therapies to inhibit its production, enhance its degradation or improve its clearance from the brain would be therapeutic [2]. One such avenue of investigation is Aβ immunotherapy. Schenk et al. demonstrated that immunizing APP transgenic mice (APP-tg) with Aβ peptide resulted in a lowering of cerebral Aβ deposition [3]. Several subsequent studies demonstrated the importance of antibody mediated clearance of Aβ and its role in improving cognition [4-7]. Recently it has been demonstrated that non-B cell mechanisms may also have a role in clearing cerebral Aβ [8]. A multi-center Aβ vaccine human clinical trial (AN1792) was initiated but was suspended when approximately 6% of the subjects experienced symptoms of meningoencephalitis [9-11]. To date, three autopsy case reports from AN1792 participants demonstrated a reduction in Aβ plaque number compared to controls [12-14]. However, a T cell infiltrate was present in the leptomeninges, perivascular spaces and parenchyma of the brain in two of the cases, suggesting a T cell mediated immune response to the vaccination. In the other report, there was little evidence of overt inflammation at the time of death [14] however, this does not rule out that inflammation had been present but resolved by the time of autopsy. Therefore, Aβ based immunotherapy has potential but more research is required to determine why a subset of patients experienced adverse outcomes.

We and others have demonstrated that the B cell epitope in humans [15], monkeys [16] and mice [17-19] is located in the Aβ1–15 region, whereas the T cell epitope has been mapped to within Aβ15–42 [20,21]. Based on these non-overlapping epitopes, fragments of Aβ spanning the B cell epitope but not the T cell epitopes may avoid a deleterious cellular immune response. Prior reports have suggest that this may be true as shorter Aβ fragments conjugated to T cell helper epitopes [22] or mutated Aβ [23,24], have lead to a humoral immune response. Recent reports using multiple antigen peptides (MAP) have demonstrated that Aβ fragments on a branching lysine tree results in a humoral immune response [18,25]. We have previously demonstrated that immunization using Aβ1–15 peptide plus the adjuvant LT(R192G) as both the priming and boosting immunogen does not induce a humoral immune response. However, when Aβ1–15 was given intranasally as a boosting immunogen following a priming injection of Aβ1–40/42, specific Aβ antibodies were detected in wildtype B6D2F1 mice [26]. To date there has been little data regarding the T cell response to various Aβ immunogens, which is important for the design of a safer AD vaccine.

The purpose of these experiments was to determine if a single subcutaneous injection of full length Aβ plus the adjuvant LT(R192G) (priming dose) followed by multiple intranasal boosts with either dendrimeric Aβ1–15 (dAβ1–15, 16 copies of Aβ1–15 on a branching lysine tree), Aβ1–15 peptide (a single copy of Aβ1–15) or Aβ1–40/42 would result in a humoral immune response. The main advantage of the prime/boost strategy is the increased immune response due to the priming with the full-length peptide and then focusing the immune response to a specific region by boosting with a smaller peptide. The use of the whole peptide increases the chances that the immune system will recognize the peptide and initiate the immune response. The ability of these vaccination strategies to avoid a T cell response was measured using splenocyte proliferation assays and cytokine specific ELISAs. The efficacy of the different immunogens plus the adjuvant LT(R192G) to reduce cerebral Aβ and the attending pathology was examined.

## Methods

### Animals

Heterozygous J20 APP-tg mice (APP_sw _and _V717F_) (C57BL/6 X DBA2 background) [27] and non-transgenic littermates were from our breeding colony and vaccination was begun at 4.0 ± 0.1 months of age. Mice were genotyped using PCR. All animal use was approved by the Harvard Standing Committee for Animal Use and in compliance with all state and federal regulations

### Immunization

All peptides used in these studies, Aβ1–40, Aβ1–42, Aβ1–15 and dAβ1–15, were synthesized by the Biopolymer Laboratory, Center for Neurologic Diseases (Boston, MA). Aβ1–15 peptide is a single copy of the first 15 amino acids of Aβ, whereas dAβ1–15 consists of 16 copies of the same peptide on a lysine backbone. The peptides were diluted in distilled water at 4 mg/ml. For the full length Aβ1–40/42 immunogen, a mixture of Aβ1–40 (3 mg/ml) and Aβ1–42 (1 mg/ml) in distilled water was incubated overnight at 37°C. Synthetic Aβ1–42 assembles into a variety of structures in aqueous buffers, including low n-oligomers, ADDLs, protofibrils and fibrils [28]. The solutions of synthetic Aβ used in this study probably contained a mixture of these assemblies, but biophysical analysis was not performed to determine the presence or relative abundance of these species. Circular dichroism analysis of the dAβ1–15 peptide demonstrated a random structure, without α or β sheet structures (data not shown). The immunogens were aliquotted and frozen at -20°C. The adjuvant, 5 μg mutant heat labile E.coli enterotoxin, LT(R192G) (kind gift of J. Clements, Tulane University School of Medicine, New Orleans, LA) [29], was mixed with immunogen just prior to immunization.

Mice were given a priming dose of vaccine by a single s.c. injection composed of 75 μg Aβ40 and 25 μg Aβ42 mixed with 10 μg LT(R192G). Vehicle control mice received LT(R192G) alone. Boosting by intranasal vaccination was performed on a weekly basis as previously reported [30]. Briefly, 100 μg of immunogen plus 5 μg LT(R192G) was mixed and applied to the naris in 2 separate 15 μl doses, allowing capillary action to draw the liquid into the nasal cavity. Control mice received LT(R192G) alone. All vaccinations were administered weekly for 6 months.

### Plasma and tissue collection

Blood was collected from the tail bi-weekly and the plasma frozen at -20°C. One week following the final immunization, mice were sacrificed by CO_2 _inhalation. Blood was collected by cardiac puncture followed by perfusion with 10 ml Tris buffered saline (TBS). The spleen was aseptically removed and placed in RPMI on ice for cell culture studies. The brain was removed and divided sagittally into two hemispheres. One hemi-brain, as well as pieces of liver, kidney and spleen was placed in 10% buffered neutral buffered formalin for 2 hours, processed, and embedded in paraffin. The other hemi-brain was snap frozen and stored at -80°C for biochemical analysis of Aβ.

### Anti-Aβ antibody ELISA

Anti-Aβ antibodies in plasma were measured by ELISA as previously described [30]. ELISAs for antibody isotypes and epitope mapping were performed as previously reported [31]. Briefly, quantitative Ig isotype-specific ELISAs for IgG1, IgG2a, IgG2b, IgA and IgM anti-Aβ antibodies were performed by adding a standard curve of the appropriate Ig isotype (Southern Biotechnology Associates, Birmingham, AL) to the standard immunoassay and using biotinylated isotype specific secondary antibodies (Zymed, San Francisco, CA). Peptide competition assays to determine antibody B cell epitopes were performed as previously described [32]. The following overlapping Aβ fragments (CND Biopolymer Laboratory) were used for antibody epitope mapping; Aβ1–15, Aβ1–7, Aβ3–9, Aβ6–20, Aβ11–25, Aβ26–42, and Aβ1–40. Diluted plasma samples were co-incubated with peptide fragments overnight and applied to Aβ1–40-coated ELISA plates.

### Immunohistochemistry and image analysis

Immunohistochemistry was performed on 12 μm paraffin sections using the ELITE ABC method (Vector Laboratories, Burlingame, CA) as previously described. [33]. The following antibodies and dilutions were used to examine T cells (CD5, 1:50; BD PharMingen, San Jose, CA), B cells (CD45RC, 1:500; BD PharMingen), activated microglia/macrophage, (CD45, 1:5000; Serotec, Raleigh, NC) or astrocytes (GFAP, 1:1000; Dakocytomation, Carpinteria, CA). Rabbit polyclonal Aβ antibodies DW14 1:1000 and R1282 1:1000 (gifts of D. Walsh and D. Selkoe, respectively, Center for Neurologic Diseases, Boston, MA) were used to visualize Aβ deposition. Positive controls (sections of spleen and brain from mice with experimental autoimmune encephalitis and aged APP-tg mice) and negative controls (normal immunoglobulin) were included. To screen plasma for antibody binding to AD plaques, paraffin-embedded human AD brain tissue was used as previously reported [34].

The Perl's Prussian blue method of hemosiderin staining was performed to examine brains for ferric iron (found in hemaglobin) as a measure of hemorrhage. Briefly, de-waxed sections were submerged in a solution of 2% potassium ferrocyanide and 2% HCl for 20 minutes, followed by rinsing and eosin counterstaining.

Images were captured for quantification from 4–6 sections of hippocampus using a 4× objective for R1282 staining (Aβ) or a 10× objective for CD45 (microglia) and GFAP (astrocytes). Acquisition of images was performed in a single session using a SPOT camera (Sterling Heights, MI). Computer-assisted image analysis was performed using IP Lab Spectrum 3.1 Image Analyzer software (Fairfax, VA), with the hippocampus as the region of interest (ROI).

### Splenocyte cultures

All cell culture reagents were from Invitrogen (Los Angeles, CA). Splenocytes were isolated and harvested using standard methods as previously reported [32]. Aβ peptides were added to cultures in triplicate at a final concentration of 0, 0.5, 5 or 50 μg/ml. At 48 and 72 hours, supernatants were collected and analyzed by ELISA for cytokines. To measure proliferation, 1 μCi of [^3^H]-thymidine was added to cells at 72 h. Eighteen hours later cells were harvested and thymidine incorporation determined using a liquid scintillation counter. A stimulation index was calculated using the following formula: CPM of well with antigen/CPM with no antigen.

### Cytokine ELISA

Cytokine levels were measured in splenocyte supernatants using matching antibody pairs composed of a capture and detection antibody for IL-4 and IFN-γ (BD PharMingen).

### Aβ ELISA

Both soluble and insoluble brain Aβ levels were determined. For soluble Aβ levels, frozen hemibrains were homogenized in 4 volumes of TBS with a protease inhibitor cocktail (Sigma, Saint Louis, MO)). The samples were centrifuged at 60,000 RPM for 30 minutes at 4°C. The supernatant was collected and stored at -20°C. TBS insoluble Aβ protein was extracted as previously described [35] using 10 volumes of ice cold guanidine buffer (5 M guanidine-HCl/50 mM Tris, pH 8.0). ELISAs specific for human Aβ_40_, Aβ_42 _andtotal Aβ were performed (using antibodies kindly supplied byELAN Pharmaceuticals) as previously described [36].

### Statistical analysis

Kruskal-Wallis nonparametric one-way ANOVA analysis was used to determine statistical significance between groups using Prism version 4.0 (GraphPad Software, San Diego, CA).

## Results

### Immunization with dAβ1–15 or Aβ1–40/42 but not Aβ1–15 peptide results in anti-Aβ antibody production in APP-tg and wildtype mice

**Figure 1:**
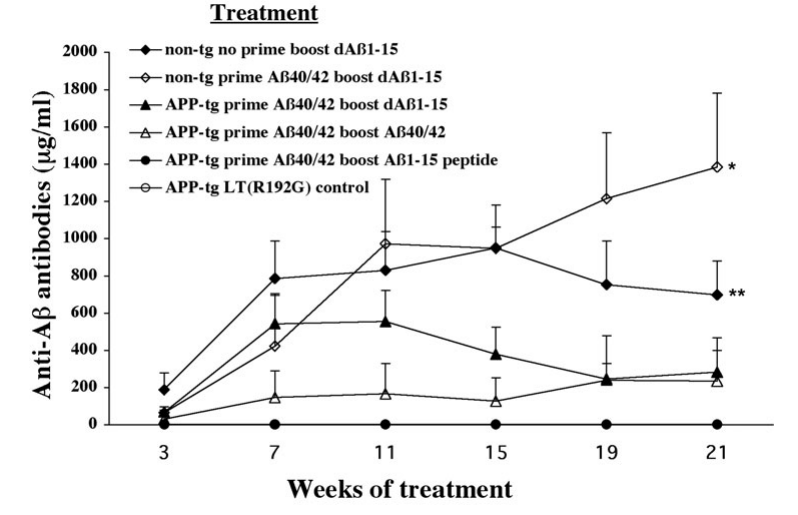
**Anti-Aβ antibodies are produced by priming with Aβ1–40/42, followed by boosting with Aβ40/42 or dAβ1–15**. J20 APP-tg mice primed with a single subcutaneous dose of Aβ1–40/42 with LT(R192G) and subsequently boosted intranasally with Aβ1–40/42 (n = 6) or dAβ1–15 (n = 7) produce anti-Aβ antibodies. However, J20 APP-tg mice receiving adjuvant alone (n = 6) or boosted with Aβ1–15 peptide (n = 8) did not produce any detectable antibodies. Wildtype littermates immunized with a priming dose of Aβ1–40/42 and boosted with dAβ1–15 (n = 4) generated significantly higher levels of anti-Aβ antibodies compared to any of the immunized J20 APP-tg mice regardless of treatment group (*p < 0.05, Kruskal-Wallis one-way ANOVA). Wildtype mice immunized by dAβ1–15 alone (n = 4) produced high levels of anti-Aβ antibodies that were significantly greater than those produced by APP-tg mice primed with Aβ1–40/42 and boosted with Aβ1–15 and vehicle controls (**p < 0.05, Kruskal-Wallis one-way ANOVA). Mean ± SEM.

Using a specific ELISA the levels of plasma anti-Aβ antibodies were measured throughout the period of immunization. Anti-Aβ antibodies were detected after only 3 treatments in mice receiving either dAβ1–15 or full length Aβ1–40/42 after a priming dose of full length Aβ1–40/42 (Figure 1). No mice receiving either LT(R192G) adjuvant alone or Aβ1–15 peptide produced antibodies, even though the Aβ1–15 peptide group received a priming dose of Aβ1–40/42. Interestingly, the immunized wildtype mice produced greater amounts of anti-Aβ antibodies compared to their APP-tg littermates. All wildtype mice produced antibodies in response to immunization, whereas anti-Aβ antibodies were not detected in a small percentage of APP-tg mice (Table 1). Plasma from mice producing anti-Aβ antibodies as measured by ELISA bound Aβ plaques in cerebral tissue obtained from AD subjects (data not shown).

**Table 1:** Percentage of responders and Aβ antibody isotypes

Strain	Immunogen	Prime (Y/N)	Responder^	IgG1*	IgG2a*	IgG2b*	IgM*	IgA*
WT	dAβ1–15	Y	4/4	265 ± 89	71 ± 47	257 ± 130	68 ± 22	116 ± 35
WT	dAβ1–15	N	4/4	238 ± 105	77 ± 49	742 ± 178	86 ± 21	122 ± 30
APP-tg	dAβ1–15	Y	5/7	148 ± 48	75 ± 59	214 ± 99	106 ± 15	46 ± 11
APP-tg	Aβ40/42	Y	5/6	45 ± 27	50 ± 48	234 ± 103	76 ± 21	66 ± 17

^ number of mice with detectable plasma anti-Aβ antibodies/total number mice immunized

* μg/ml ± SEM; Y = yes, N = no.

**Figure 2:**
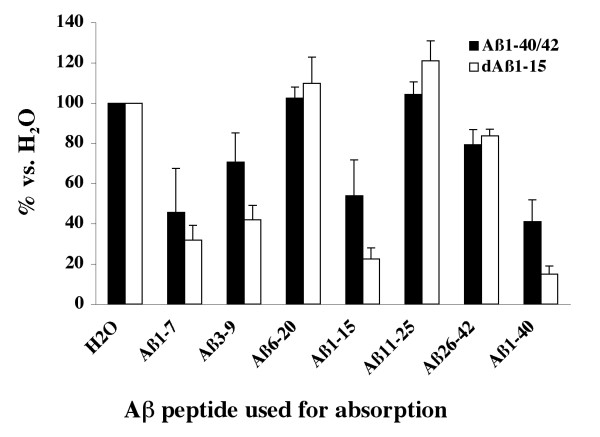
**Anti-Aβ antibodies recognize an epitope within the Aβ1–7 region**. Absorption of diluted plasma from J20 APP-tg mice producing anti-Aβ antibodies with Aβ1–7, Aβ1–15 or Aβ1–40 peptide reduced binding to plate-bound Aβ, thereby demonstrating that the antibodies recognized an epitope within the Aβ1–7 region.

Isotypes were examined using specific secondary antibodies in ELISAs. There was a mixture of IgG1, IgG2b, IgG2a and IgA anti-Aβ antibodies in all mice that generated antibodies in response to vaccination (Table 1). However, IgG2b and IgG1 were the predominant isotypes. Epitope mapping demonstrated that the antibodies bound a region located in the 1–7 region of Aβ, regardless of the immunogen used (Figure 2).

### Limited anti-Aβ cellular immune response found in Aβ vaccinated J20 APP-tg and wildtype mice

**Figure 3:**
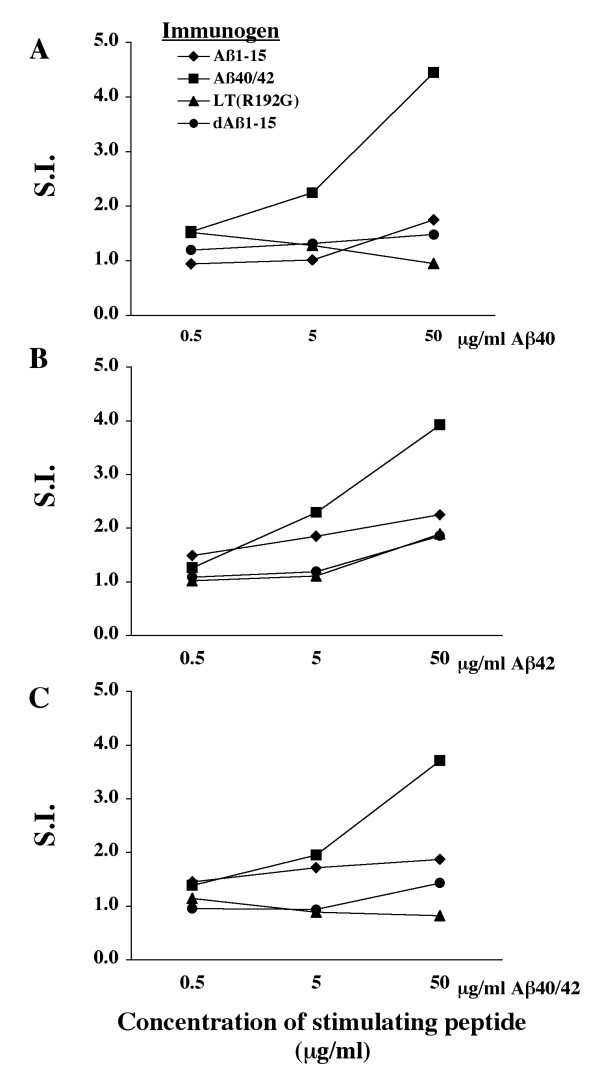
**Minimal T cell reactivity to Aβ peptides in dAβ1–15 immunized J20 APP-tg mice**. Splenocytes were isolated, pooled into treatment groups and stimulated with Aβ1–40 (A), Aβ1–42 (B) or a combination of Aβ1–40/42 (C) in triplicate. Following 72 hours of incubation, 3[H] thymidine was added and the radioactivity measured after 18 hours. Mice primed by s.c. injection of Aβ1–40/42 and boosted intranasally with Aβ1–40/42 proliferated more than mice in the other treatment groups. J20 APP-tg mice boosted with LT(R192G), Aβ1–15 peptide or dAβ1–15 did not proliferate to any of the peptides (S.I. <2).

Splenocytes were isolated from all J20 APP-tg mice and re-stimulated in vitro with either Aβ1–40, Aβ1–42 or a mixture of Aβ1–40/42. All peptides were incubated for 24 hours at 37°C to allow fibrils to form. Considering a S.I. above 2 to be proliferation above background, only J20 APP-tg mice immunized with Aβ1–40/42 showed splenocyte proliferation after re-stimulation with full length Aβ peptide, indicating a T cell immune response (Figure 3). Mice immunized with dAβ1–15, Aβ1–15 peptide or LT(R192G) alone did not show a significant splenocyte proliferation (S.I. < 2.0) Cultured splenocytes were re-stimulated with either Aβ1–40, Aβ1–42 or Aβ1–40/42 to determine if the species of Aβ had an effect on splenocyte proliferation. There was no significant difference in the proliferation when the different peptides were compared. Using specific and sensitive ELISAs, IFN-γ and IL-4 were not detected in the splenocyte culture supernatants when stimulated with any of the tested peptides.

**Figure 4:**
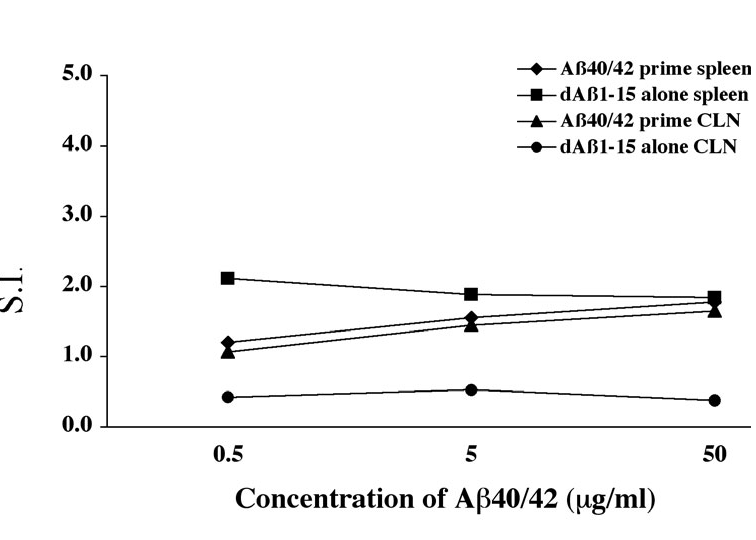
**No T cell reactivity in dAβ1–15 immunized wildtype mice**. Cells from the spleen and cervical lymph nodes were isolated from B6D2F1 mice that received either a prime injection with Aβ1–40/42 and subsequent boosting intranasally with dAβ1–15 or received intranasal dAβ1–15 alone. The cells were pooled with respect to treatment group and tissue and stimulated in vitro with a mixture of Aβ1–40/42. There was no significant proliferation of cells from either tissue, regardless of the treatment (S.I. <2), thus indicating no population of anti-AβT cells in the draining lymph nodes of the nasal mucosa.

The cellular immune response in dAβ1–15 treated wildtype littermates was also examined. No splenocyte proliferation was detected in the group receiving a priming injection of Aβ40/42, followed by intranasal boosting with dAβ1–15 or those mice receiving i.n. dAβ1–15 alone (Figure 4). In addition, cervical lymph nodes were cultured to examine whether these draining lymph nodes contained Aβ reactive lymphocytes. Similar to the splenocyte cultures, no proliferation was observed (Figure 4). IFN-γ and IL-4 were not detected in the supernatants from either the splenocyte or lymph node cultures.

### Immunization of J20 APP-tg mice reduces cerebral Aβ plaques and attending pathology

**Figure 5:**
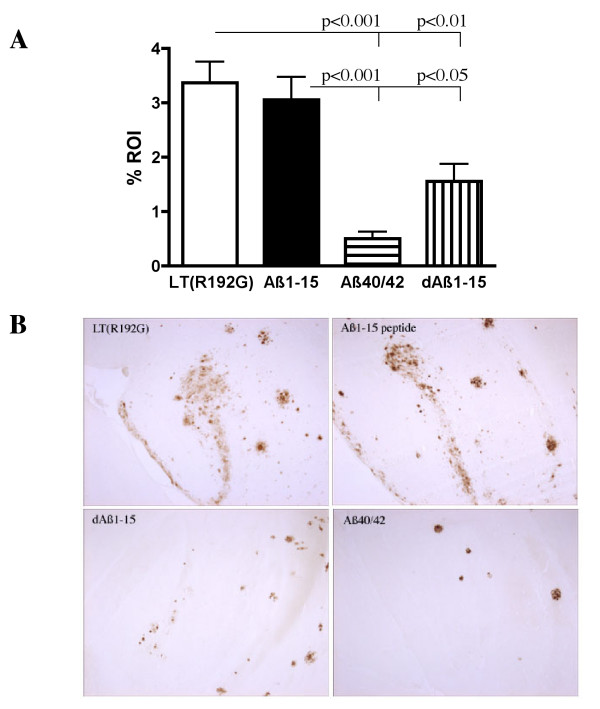
**Prime/boost immunization of J20 APP-tg mice with Aβ1–40/42 or dAβ1–15 leads to a reduction in hippocampal Aβ plaque burden**. Following a 6-month immunization regime, the cerebral tissue was harvested, immunohistochemistry performed and computer assisted quantification completed. J20 APP-tg mice primed with an injection of Aβ1–40/42 and boosted with Aβ1–40/42 or dAβ1–15 had significantly less hippocampal area covered by Aβ immunoreactivity compared to LT(R192G) treated controls and mice boosted with Aβ1–15 peptide (A). Each group is composed of the mean of 2–6 sections from 5–7 different mice in each treatment group. Statistical significance was determined using Kruskal-Wallis nonparametric one-way ANOVA. (B) Immunostaining with the anti-Aβ rabbit polyclonal antibody, R1282, demonstrates a reduction in cerebral Aβ plaques. Vehicle control mice and mice boosted with Aβ1–15 peptide had mostly diffuse Aβ deposits with a small number of compacted plaques in the hippocampus at 10 months of age. However, in mice boosted with dAβ1–15 or Aβ1–40/42 diffuse Aβ was absent and somewhat fewer compacted Aβ plaques remained in the hippocampus, following 6 months of treatment. Scale bar = 100 μm.

Computer-assisted quantification of immunohistochemistry was used to examine the hippocampus of 10 month-old J20 APP-tg mice following 6 months of immunization. Mice that did not produce anti-Aβ antibodies (i.e., "non-responders") were excluded from this analysis, as the purpose of these experiments was to determine if successful treatment would decrease cerebral Aβ and its attending pathology. Immunohistochemistry using polyclonal anti-Aβ antibodies demonstrated a significant decrease in the Aβ plaque burden in those mice receiving a subcutaneous prime of Aβ1–40/42, followed by i.n. boosting with Aβ1–40/42 or dAβ1–15 (Figures 5). This decrease was not seen in the groups of J20 APP-tg mice receiving Aβ1–15 peptide as a boosting immunogen.

To examine the glial response to immunization, GFAP and CD45, markers for reactive astrocytes and activated microglia respectively, were examined. At 10 months of age, limited numbers of compacted plaques and very few activated microglia were observed in hippocampus of non-immunized J20 APP-tg mice. For example, CD45 immunoreactivity occupied only 0.56 ± 0.19% of the hippocampus area. There were no significant differences between any of the treatment groups for either microglial labeling with anti-CD45 or astrocyte labeling with anti-GFAP (data not shown), most likely due to the low numbers of compacted plaques at this age.

To determine if autoimmune encephalitis was induced by the immunization protocols used in these experiments, the brain was examined for the presence of lymphocytes. No T or B cells were found in any regions of the brain. Additionally, no instances of micro-hemorrhages were found as examined by hemosiderin staining.

To confirm the decrease in cerebral Aβ detected immunohistochemically, biochemical analysis was performed on J20 APP-tg mice using specific ELISAs for Aβx-40 and Aβx-42. There was a non-significant reduction of Aβ40 and Aβ42 TBS and guanidine soluble levels (commonly referred to as soluble and insoluble fractions) in both the Aβ1–40/42 and dAβ1–15 boosted J20 APP-tg groups, compared to the LT(R192G) and Aβ1–15 peptide groups (Table 2). Plasma levels of total Aβ were elevated in the J20 APP-tg mice boosted with Aβ1–40/42 compared to the other treatment groups.

**Table 2:** Cerebral and plasma Aβ levels in J20 APP-tg mice

	TBS soluble*		Guanidine soluble*		Plasma^
	
Treatment group†	Aβx-40	Aβx-42	Aβx-40	Aβx-42	Aβ1-total
LT(R192G)	8.1 ± 1.8	12.3 ± 1.1	485.8 ± 123.0	2017.5 ± 659.1	370 ± 29
Aβ40/42	6.0 ± 1.8	10.1 ± 3.0	178.1 ± 49.8	757.6 ± 186.7	522 ± 81
Aβ1–15	9.4 ± 1.6	13.3 ± 1.9	331.7 ± 71.2	2024 ± 829.9	316 ± 46
dAβ1–15	5.8 ± 1.9	7.8 ± 2.5	224.1 ± 58.9	1345 ± 551.5	344 ± 86

* ng/ml

^ pg/ml

† all mice except the LT(R192G) control mice received a priming dose of Aβ40/42 before the boosting doses of the immunogens listed in this table

## Discussion

These experiments were performed to examine if a priming and boosting vaccination protocol using different immunogens would lead to an effective humoral immune response and a clearing of cerebral Aβ in an APP-tg mouse model. This strategy has been successful in the induction of a cellular immune response [37], however we were interested if this same immunization regime could be utilized to induce a humoral immune response. We report that an initial injection of full length Aβ1–40/42 (priming), followed by subsequent weekly intranasal immunization with either Aβ1–40/42 or dAβ1–15 (boosting) led to a humoral immune response. However, no anti-Aβ antibodies could be detected in the plasma of mice primed with Aβ1–40/42 and boosted with Aβ1–15 peptide. This is in contrast to our previous report using wildtype B6D2F1 mice, which are a similar genetic background as the J20 APP-tg mice used in the present study [26]. In our previous report, we demonstrated that i.n. boosting with Aβ1–15 peptide following an intraperitoneal priming injection of Aβ1–40/42 resulted in a long lasting humoral immune response. The difference between studies may be due to the intrinsic immune tolerance seen in the J20 APP-tg mice. These mice, unlike their wildtype littermates and B6D2F1 mice, are exposed to human Aβ throughout their lives due to their transgene expression. Therefore, this protein is considered a self-antigen and requires the breaking of immune tolerance to generate anti-Aβ antibodies. In addition, this is likely the phenomena responsible for the lower anti-Aβ antibodies seen in the J20 APP-tg mice compared to their wildtype littermates in the present experiment. Regardless of the mechanism it appears that a combination of Aβ1–40/42 priming and i.n. boosting with Aβ1–15 peptide does not result in a humoral immune response in J20 APP-tg mice.

Mechanisms to overcome immune tolerance include the use of adjuvants and presenting the peptide in a novel manner. The use of the adjuvant LT(R192G) was not enough to overcome tolerance as seen with the lack of a immune response to Aβ1–15 peptide. Therefore, we constructed a novel immunogen, dAβ1–15 and tested it in both wildtype and J20 APP-tg mice. Dendrimeric immunogens have the unique ability to present multiple copies of the peptide and are larger molecules compared to a single copy of the peptide [38-40]. These two properties allow the immunogen to be longer lived, thus increasing its potential to be phagocytosed, presented by antigen presenting cells and allowing an immune response to be generated. This is the likely the reason full length fibrillar Aβ can induce a humoral immune response in humans [41,42], monkeys [16] and mice [3]. Dendrimeric Aβ1–15 when utilized as a boosting immunogen induced anti-Aβ antibodies in a similar amount as boosting with full length Aβ1–40/42. Indeed, in wildtype littermates, a priming injection of Aβ1–40/42 was not required to stimulate a humoral immune response. In all immunized groups, regardless if the mice received Aβ1–40/42 or dAβ1–15, the antibodies were directed to an epitope found in the Aβ1–7 region, similar to other studies from our laboratory [16,32,36,43] and others [18,20,22,42]. Therefore, it appears that dAβ1–15, unlike Aβ1–15 peptide, is an effective boosting immunogen in J20 APP-tg mice. The higher number of immunogens and its repetitive structure may allow dAβ1–15 to overcome tolerance in humans. For an AD vaccine to be successful, it should induce a humoral immune response in the majority of subjects, which was not the case in the AN1792 trial, as many subjects did not produce anti-Aβ antibodies [44]. The use of novel immunogens, including dAβ1–15, may help overcome this problem.

The construction of dAβ1–15 includes the B cell epitope but avoids the reported T cell epitope [18,21]. As a T cell mediated immune response is hypothesized to be the basis of the meningoencephalitis reported in the AN1792 trial [10], this may be an important consideration in the design of a safe, effective vaccine. A strong humoral immune response was found in both wildtype and J20 APP-tg mice, following i.n. boosting with either Aβ1–40/42 or dAβ1–15. Splenocyte cultures demonstrated that there was no proliferation in response to stimulation with Aβ1–40, Aβ1–42 or Aβ1–40/42 in dAβ1–15 immunized APP-tg or wildtype mice. To determine if a small population of T cells was present, we cultured the cervical lymph nodes, known to be the draining lymph nodes of the nasal mucosa. Therefore, i.n. immunization may enrich Aβ-reactive T cells in these lymph nodes compared to the spleen. However, there was no proliferation or cytokine production detected in these cultures, similar to that seen in the spleen. Interestingly, in cervical lymph nodes in mice not primed with Aβ1–40/42 but receiving only i.n. dAβ1–15, the stimulation index was below 1. One explanation for this is the induction of regulatory T cells following i.n. dAβ1–15, which may suppress Aβ reactive T cells, as shown in studies with other peptides [45,46]. Further research is required but this may be an important mechanism to reduce the induction of effector T cells. In contrast, J20 APP-tg mice receiving Aβ1–40/42 as both the priming and boosting immunogen, demonstrated a moderate proliferation of splenocytes in response to Aβ1–40, Aβ1–42 or their combination. Together these data demonstrate that dAβ1–15 boosting, subsequent to a priming dose of Aβ1–40/42, results in a humoral but not a T cell response in J20 APP-tg and wildtype mice.

The effects of i.n. boosting with dAβ1–15, Aβ1–15 and Aβ1–40/42 on the cerebral levels of Aβ was investigated in J20 APP-tg mice. Plaque burden was significantly reduced in the mice receiving dAβ1–15 and Aβ1–40/42 as compared to LT(R192G) and Aβ1–15 peptide immunized mice. This is not surprising as the former treatments resulted in the production of anti-Aβ antibodies, whereas no anti-Aβ antibodies could be detected in the latter groups. A reduction was also noted in the biochemical levels of both TBS and guanidine soluble fractions of Aβ in those groups producing Aβ specific antibodies, though it did not reach significance. The lack of a significant decrease was likely due to the variability in the Aβ protein levels in J20 APP-tg mice found at 10 months of age, as noted by the large standard error values seen in Table 2, and a dilution effect observed when the entire hemibrain is homogenized. The reduction in hippocampal Aβ plaque burden is in agreement with other reports demonstrating that immunization of APP-tg mice reduced cerebral Aβ plaque burden [3,24,36]. Reactive microglia and activated astrocytes were not significantly different between treatment groups and vehicle controls. This is likely attributable to the low numbers of compacted plaques and activated microglia detected in non-immunized J20 APP-tg mice at 10 months of age. Interestingly, after immunization, only compacted plaques were detected in hippocampus; diffuse Aβ deposits were not observed. The number of activated microglia was not increased in the Aβ immunized mice, indicating a lack of cerebral inflammatory reaction and correlating well with the lack of cellular immune response to Aβ in the splenocyte cultures. No instances of micro-hemorrhage, T or B cells were observed in the CNS of any of the wildtype or APP-tg mice regardless of the immunization regime, providing further evidence for the lack of a deleterious immune response in brain. These data provide further data towards the safety and efficacy of dAβ1–15 immunization as a potential Aβ vaccine, although it is acknowledged that the adverse events observed in the AN1792 trial in humans have not yet been replicated in APP-tg mice after active Aβ immunization. Therefore, improved mouse models mimicking the human adverse events are needed to better assess the safety of our new immunogens and immunization regimens.

## Conclusion

These studies are the first to explore the concept of a prime and boost Aβ immunization strategy using different Aβ immunogens in APP-tg mice. Following a single subcutaneous priming dose of full length Aβ1–40/42, intranasal boosting with a peptide composed of a single copy of Aβ1–15 peptide was not effective in inducing a humoral immune response in J20 APP-tg mice,. In contrast, boosting with dAβ1–15 resulted in a robust humoral immune response, with a minimal T cell response in either the spleen or draining lymph nodes. Immunization with full length Aβ1–40/42 resulted in a lowering of cerebral Aβ and a humoral immune response, but was accompanied by a modest T cell response. Taken together these data suggest that a prime/boost immunization regime with Aβ1–40/42 and dAβ1–15 may be an effective alternative compared to full length Aβ immunization in a potential AD vaccine.

## Competing interests

Cynthia Lemere has a research contract from ELAN/Wyeth Pharmaceuticals to study Aβ immunotherapy in non-human primates. The other authors have no competing interests.

## Authors' contributions

TJS performed animal treatments, collected plasma, performed necropsies, immunohistochemistry and ELISAs, and guided or performed image analysis, analyzed data and drafted the manuscript. LJ performed tissue preparation, immunohistochemistry, image analysisand ELISAs. KT carried out ELISAs and immunohistochemistry. CAL developed the design of the study, analyzed data, guided study execution, edited the manuscript, and submitted it online for publication. All authors read and approved the final manuscript.
